# Potential enhancement of host immunity and anti-tumor efficacy of nanoscale curcumin and resveratrol in colorectal cancers by modulated electro- hyperthermia

**DOI:** 10.1186/s12885-020-07072-0

**Published:** 2020-06-29

**Authors:** I-Ming Kuo, Jih-Jong Lee, Yu-Shan Wang, Hsin-Chien Chiang, Cheng-Chung Huang, Pei-Jong Hsieh, Winston Han, Chiao-Hsu Ke, Albert T. C. Liao, Chen-Si Lin

**Affiliations:** 1 Department of Veterinary Medicine, School of Veterinary Medicine, National Taiwan University, 1 Sec 4 Roosevelt Road, Taipei, 10617 Taiwan; 2 Graduate Institute of Veterinary Clinical Science, School of Veterinary Medicine, National Taiwan University, Taipei, Taiwan; 3 Institute of Molecular Medicine and Bioengineering, National Chiao Tung University, Hsinchu, Taiwan; 4 JohnPro Biotech Inc., Taipei, Taiwan

**Keywords:** Modulated electro-hyperthermia (mEHT), Curcumin, Resveratrol, Nanosized, Apoptosis, Tumor microenvironment

## Abstract

**Background:**

Modulated electro-hyperthermia (mEHT) is a form of hyperthermia used in cancer treatment. mEHT has demonstrated the ability to activate host immunity by inducing the release of heat shock proteins, triggering apoptosis, and destroying the integrity of cell membranes to enhance cellular uptake of chemo-drugs in tumor cells. Both curcumin and resveratrol are phytochemicals that function as effective antioxidants, immune activators, and potential inhibitors of tumor development. However, poor bioavailability is a major obstacle for use in clinical cancer treatment.

**Methods:**

This purpose of this study was to investigate whether mEHT can increase anti-cancer efficacy of nanosized curcumin and resveratrol in in vitro and in vivo models. The in vitro study included cell proliferation assay, cell cycle, and apoptosis analysis. Serum concentration was analyzed for the absorption of curcumin and resveratrol in SD rat model. The in vivo CT26/BALB/c animal tumor model was used for validating the safety, tumor growth curve, and immune cell infiltration within tumor tissues after combined mEHT/curcumin/resveratrol treatment.

**Results:**

The results indicate co-treatment of mEHT with nano-curcumin and resveratrol significantly induced cell cycle arrest and apoptosis of CT26 cells. The serum concentrations of curcumin and resveratrol were significantly elevated when mEHT was applied. The combination also inhibited the growth of CT26 colon cancer by inducing apoptosis and HSP70 expression of tumor cells while recruiting CD3+ T-cells and F4/80+ macrophages.

**Conclusions:**

The results of this study have suggested that this natural, non-toxic compound can be an effective anti-tumor strategy for clinical cancer therapy. mEHT can enable cellular uptake of potential anti-tumor materials and create a favorable tumor microenvironment for an immunological chain reaction that improves the success of combined treatments of curcumin and resveratrol.

## Background

Modulated electro-hyperthermia (mEHT), a form of hyperthermia treatment [1–3], heats tissue via capacitive-impedance coupled 13.56 MHz amplitude-modulated radiofrequency [1]. mEHT selectively forwards energy to the most ionized areas within the tumor as well as the surrounding microenvironment, allowing heating to specifically target tumors during treatment. The cell membrane is an important target for mEHT [4]. The cell membrane rafts, which consist of a cluster of functional proteins, have different electromagnetic properties as compared with other parts of the cell membrane, thereby making membrane rafts absorb more mEHT energy than other lipid bilayer parts of the cell membrane. Energy absorption leads to temperature increase of cell membrane rafts, consequently disrupting membrane arrangement and integrity, leading to enhanced cellular uptake of liposomal drugs [5].

mEHT centers radiofrequency on tumor tissues, and the energy absorbed results in temperature elevation to fever-like range (at or below 42 °C), thus inducing apoptosis of tumor cells [2, 6]. Furthermore, mEHT also triggers the release of heat shock protein 70 (Hsp70) by tumor cells [7]. The overexpression and release of Hsp70 has been proven to be positively associated with favorable prognosis and can activate innate immunity [8].

mEHT has been used clinically in several cancer types, including breast, ovarian, and cervical [9, 10]. In the clinical setting, mEHT is recommended to be used in combination with radiotherapy, chemotherapy, or immunotherapy, to increase efficacy [11, 12]. When combined with mEHT, chemotherapy was found to have increased cellular uptake, increasing cytocidal effects in cancer cells [13]. In lung carcinoma, hyperthermia treatment was shown to enhance curcumin retention, resulting in cancer cell death [14].

Curcumin is a well-known dietary polyphenol extracted from the rhizome of turmeric (*Curcuma longa*). Turmeric, an Indian spice commonly used in preparation of curry and mustard [15], is a nature antioxidant with very low toxicity [16, 17]. Curcumin is known to have anti-inflammatory, anti-microbial, antioxidant properties [18–20], and is known as a cancer chemopreventive agent in several kinds of cancers, including brain, breast, colon, head and neck, melanoma ovarian, pancreatic, and prostate [21, 22]. It has been reported that curcumin suppresses tumor development by inhibiting NF-kκB, Akt/PI3K, and MAPK pathways [22–24]. In addition, curcumin also enhances host anti-tumor immunity by mediating the restoration of T-cell populations, reversing type-2 cytokine bias, reducing the population of regulatory T-cells, and inhibiting T-cell apoptosis [25]. However, curcumin has low bioavailability due to poor aqueous solubility, which partially resulting in limited use in clinical oncology [26].

Resveratrol is a nature antioxidant widely contained in grapes, Japanese knotweed, berries, peanuts, and other plants [27]. Resveratrol has also been found to inhibit several kinds of tumors, such as though of the breast, colon, and prostate [28–30], with low toxicity and side effects [31]. It is demonstrated that resveratrol is able to induce mitochondria-mediated apoptosis in tumor cells via sirtuin and NF-ϰB signaling pathways [32]. In breast cancer, resveratrol is shown to suppress proliferation via modulating CDK4/cyclin D1 expression and increasing cytoplasmic concentration of calcium to activate p21 and p53, resulting in apoptosis of cancer cells [33, 34]. In colorectal cancer, resveratrol regulates MALAT1 to alter the nuclear localization of β-catenin, resulting in reduced Wnt/β-catenin signaling which inhibits tumor invasion and metastasis [35]. However, similar to the problem of curcumin, poor bioavailability of resveratrol is regarded as a major obstacle in clinical use for cancer treatment [36].

One study used liposome-encapsulation to increase bioavailability of resveratrol and curcumin, intensifying anti-tumor effects in prostate cancer [37]. Since mEHT can specifically target tumor tissues, induce apoptosis, attack lipid raft, and disrupt the integrity of cell membrane to enable influx of potential chemo drugs, this study intends to use mEHT to increase tumor cell uptake of curcumin and resveratrol. As both compounds have multiple anti-tumor and immuno-regulating activities, synergistic tumor-suppression effects and mechanisms will be further investigated in this study.

## Methods

### Cell culture and preparation of nanoscale curcumin and resveratrol

The mice colon cancer cell line, CT26, was provided by Johnpro Biotech (Taipei, Taiwan). The cells were maintained in ATCC-formulated RPMI-1640 Medium containing 10% heat-inactivated fetal bovine serum (FBS) and 1% antibiotic-antimycotic (GM) in a humidified incubator with 5% CO_2_ at 37 °C.

### Nano formulation of curcumin plus resveratrol

Nano-sized curcumin and resveratrol compound was prepared by Johnpro Biotech (Taipei, Taiwan). The 250 g of curcumin and 250 g of resveratrol with 4500 ml reverse osmosis water was grinded to nanocomposite by high-energy miller for 4.5 h (JBM-C020, Just Nanotech Co., Ltd., Taiwan). Particle sizes were detected by Nanotrac Wave II (Microtrac, USA), with diameter of all nanocomposites measured at roughly 320 nm.

### Animal treatment and sample preparation

Male Sprague-Dawley rats weighing 241 to 247 g were purchased from BioLASCO (Taipei, Taiwan). Animals were acclimated with regular rat feed and drinking water ad libitum for 2 to 5 days before the study. Rats were administered 300 mg/kg of curcumin suspension, curcumin nanoparticles, resveratrol suspension, and resveratrol nanoparticles by oral gavage, respectively. Serial blood samples (~ 150 μL/each) were collected from all animals through the tail veins. Blood samples were collected at pre-dose, 15 min, 30 min, 1 h, 2 h, 4 h, 8 h, and 24 h post-dose. Curcumin and resveratrol were found to be unstable in rat plasma. To stop the potential degradations in rat blood, the blood samples once drawn from rats were immediately mixed with acetonitrile in a ratio of 1:8 (v/v). The deproteinized samples were temporarily held on ice, followed by storage at − 80 °C before bioanalysis. Analysis of blood concentrations were determined by LC/MS/MS. The blood-acetonitrile mixtures were vortex-mixed briefly at high speed and were then centrifuged at 20,000×g for 5 min. Approximately 50 μL of the supernatant of each mixture was transferred to a clean autosampler vial with insert for analysis. A 5 μL aliquot of each supernatant was subsequently injected into the LC/MS/MS system. Standards and quality controls were included with samples for the run so that intraday and inter-day variability was adjusted with the standards.

### Chromatographic and mass spectrometric specifications

LC/MS/MS analyses were performed on an Agilent LC 1200 HPLC System (Agilent Technologies, USA) coupled to a mass spectrometer with turbo electrospray ion source (QTrap5500 System, ABI Sciex, Canada). In both curcumin and resveratrol, analysis methods by mass spectrometer utilized an electrospray ionization (ESI) source in negative ion mode, with multi-reaction monitoring (MRM). Chromatographic separation was achieved with gradient elution on a Poroshell 120 EC-C18 column (2.7 μm; 3.0 × 50 mm, Agilent Technologies). The sample injection volume was 5 μL, and the total run times were 3 min and 2.5 min for curcumin and resveratrol, respectively. The transition (precursor to daughter) monitored in curcumin method was m/z 367.1 → 217, and in resveratrol method was m/z 227.1 → 185. The multi-reaction monitoring (MRM) data was acquired and the chromatograms were integrated using Analyst (ver. 1.5.2) software (Applied Biosystems, USA). Weighted linear regressions were used to generate the calibration curves from standards (curcumin and resveratrol) and to calculate the sample concentrations.

### Pharmacokinetic data analysis

The pharmacokinetic parameters of curcumin and resveratrol were analyzed by noncompartmental analysis using Phoenix™ for WinNonlin Program, version 6.3 (Phoenix WinNonlin, Pharsight Corporation, Mountain View, CA). Pharmacokinetic results were represented as mean ± SEM.

### Cell viability assay

CT26 cells were seeded in a 96-well plate with 1.2 × 10^4^ per well and treated with the indicated concentration of curcumin (Merck, Germany), resveratrol (Sigma-Aldrich, USA), or curcumin and resveratrol combined. DMSO and EtOH were solvents for curcumin and resveratrol, respectively. The cell viability was determined through WST1 assay (Roche, Germany) after the 24-h treatment. The synergistic effects of combined usage of curcumin and resveratrol was analyzed by CompuSyn software (ComboSyn, USA).

### Cell cycle analysis

CT26 cells were seeded into a 6-well plate with 2.4 × 10^5^ per well and treated with the indicated concentration of curcumin, resveratrol, or both for 24 h. The cells were harvested and washed with ice-cold phosphate-buffered saline (PBS) solution twice. Vortexed gently, ice-cold 70% EtOH for fixation of the sample lysate was gradually added. The cells were stored at the − 20 °C refrigerator for at least 1 day. The pellet was re-suspended in PBS and washed with PBS twice. Incubated samples with 10 μg/mL DNase-free RNase A (Sigma-Aldrich, USA) and 83 μg/mL propidium iodide (Sigma-Aldrich, USA) at 37 °C for 30 min. The cell-cycle distribution was analyzed by flow cytometry (BD Accuri™) with C6 software (BD Biosciences, USA).

### In vitro hyperthermia treatment using water bath

The CT26 cells (2 × 10^6^) were placed in a 15 ml centrifuge tube and incubated in the laboratory water bath at a serial increase of the water temperature from 30 °C, 37 °C, and 42 °C, with each incubation lasting 5 min. Another 25-min bath at 42 °C water were performed on cells for the apoptosis analysis.

### In vitro hyperthermia treatment using mEHT

Electromagnetic wave heating was provided using a capacitively-coupled, amplitude-modulated, 13.56-MHz radiofrequency (LabEHY, Oncotherm Ltd., Germany). An in vitro heating model was established in an electrode chamber (LabEHY in vitro applicator). CT26 cells (2 × 10^6^) were contained within the cell bag which was settled in the electrode chamber. The cells were then heated at 42 °C for 30 min with an average power of 10 ~ 12 W under the monitoring by optical sensors (Luxtron FOT Lab Kit, LumaSense Technologies, USA). The in vitro model schematic diagram is illustrated in Supplementary Figure 1.

### Apoptosis assay

Annexin V–fluorescein isothiocyanate (FITC) apoptosis detection (BD Biosciences) was performed according to the manufacturer’s instructions and analyzed by the flow cytometer. CT26 cells (6 × 10^5^) were pretreated with the 37 °C incubation as the control group, and water bath, or mEHT at 42 °C for 30 min. The treated cells were then exposed to the combination of curcumin (20 μM) and resveratrol (25 μM) for 3 h or 24 h. Both early apoptotic (annexin V-positive, PI-negative) and late apoptotic (annexin V-positive and PI-positive) cells were included in cell death determinations.

### Western blot

The CT26 cell lysates from the variety of the treatments were prepared using RIPA lysis buffer for immunoblotting of Cyclin D1 (Cell Signaling Technology, #92G2), Cyclin A (Santa Cruz Biotechnology, #sc-271,645), Hsp70 (Santa Cruz Biotechnology, #SC24), Caspase-3, and cleavage form of Caspase-3 (Cell Signaling Technology, #9662S). Western blot analysis was performed as previously reported [38].

### Evaluation of calreticulin (CRT) expression

CT26 cells (6 × 10^5^) were pretreated with the 37 °C incubation as the control group, and water bath, or mEHT at 42 °C for 30 min. The treated cells were then exposed to the combination of curcumin (20 μM) and resveratrol (25 μM) for 24 h. CRT expression on the cell surface was evaluated using direct immunofluorescence analysis, in which 1 × 10^5^ cells were washed twice with fluorescence-activated cell sorter (FACS) buffer (2% FBS and 0.02% sodium azide in PBS, pH 7.4) and incubated with isotype control or Alexa-Fluor 647 anti-CRT mouse monoclonal antibody (Abcam, ab196159). Cells were then washed and stained with FITC-conjugated goat anti-mouse IgG (BD Pharmingen, San Diego, CA, USA) for 30 min. Finally, all cells were washed and suspended in FACS buffer containing 5 mg/mL propidium iodide. The surface immunofluorescence of 1 × 10^4^ viable cells was measured by flow cytometry (BD Accuri™) with C6 software (BD Biosciences, USA).

### Syngeneic mouse tumor model

**Fig. 1:**
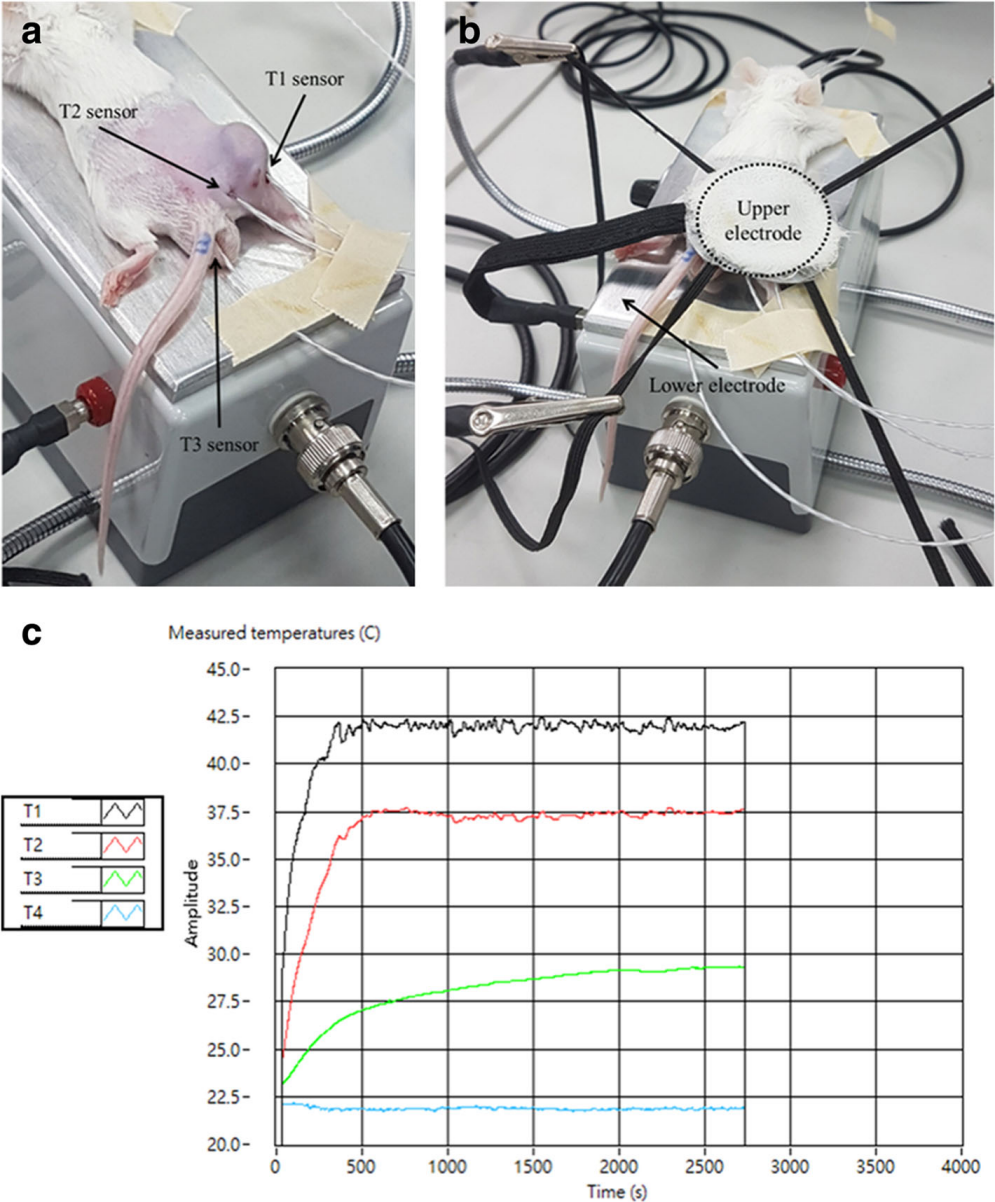
In vivo mEHT instrument. **(a)** The mouse was sedated and fixed on the mEHT instrument. The optical sensors were used to detect the temperature within the various body sites. The T1 sensor inserted into the tumor; T2 sensor inserted into the subcutaneous site; T3 sensor inserted within the rectum. **(b)** The upper electrode was covered on the tumor, which was on the wet pad to prevent from overheated. **(c)** The measured temperature curves of T1 ~ T3, T4: room temperature

Female BALB/c mice aged 6 weeks were obtained from BioLASCO. The mice were maintained in accordance with protocols approved by the Institutional Animal Care and Use Committee of National Taiwan University (IACUC No. NTU106-EL-00215). When CT26 cells were inoculated subcutaneously and the tumors reached 150 mm^3^ (length*width*width/2), mice (*N* = 6) were randomly distributed into each group including control, curcumin with resveratrol p.o. (CR), mEHT, and curcumin with resveratrol combined mEHT (CR + mEHT). The CR group mice were given curcumin 200 μg and resveratrol 105 μg every 2 days. The mEHT group mice were treated with mEHT at the first day of treatment. The CR + mEHT group mice were given both abovementioned treatments (Fig. 5a). Electromagnetic energy was generated by capacitive coupled, amplitude modulated 13.56 MHz radiofrequency (LabEHY, Oncotherm Ltd., Germany). For mice receiving mEHT, the animals were sedated with acepromazine, and fixed on the heating instrument with a single shot of mEHT for 30 min using 1 W to 3 W average power (Fig. 1a & b). To monitor temperature, optical sensors (Luxtron FOT Lab Kit, LumaSense Technologies, China) were inserted to the tumor (T1 sensor), subcutaneous site near the tumor (T2 sensor), and rectum (T3 sensor). Intratumoral temperature was kept at ~ 42 °C (+/− 0.5 °C). A rectangular grounded-aluminum electrode of 72.0 cm^2^ (kept at 37 °C) was placed below the animals and a 2.5 cm^2^ round copper-silver-tin coated flexible textile electrode was overlaid on the tumor, which was cooled under control using a wet pad. The heating temperature was maintained at ~ 42 °C (+/− 0.5 °C) while the subcutaneous temperature under the electrode was maintained at ~ 40 °C (Fig. 1c). After 14-day treatment, the mice were euthanized with isoflurane and cervical dislocation. The tumor masses on the right femoral region in each mouse were resected and the weights of whole masses were measured. Three independent experiments were conducted and the significance was analyzed in each individual test.

### Immunohistochemistry

Tumors were fixed in 10% neutral buffered formalin for 24 h and then transferred to 70% ethanol followed by processing into paraffin blocks. The blocks were then sectioned at 5 μm, followed by deparaffinization and antigen retrieval in xylene (Sigma-Aldrich, USA) at 114 ~ 121 °C for 5 min using pressure cooker in Trilogy™ (Cell Marque, USA). IHC was then performed as follows: 3% hydrogen peroxide block for 15 min, protein block (Dako) for 20 min, primary antibody incubation for 60 min [CD3 (Abcam, #ab5690), F4/80 (Cell Signaling Technology, #D259R), Hsp70 (Santa Cruz Biotechnology, #SC24)], secondary antibody incubation for 40 min (rabbit on rodent HRP polymer (Biogenex, USA), and Di-aminobenzidine (H_2_O_2_) (DAB) (Biogenex, USA) for 2.5 min. The sections were then counterstained with hematoxylin and observed under a bright-field microscope (Olympus Corporation., Japan). The number of CD3-positive, F4/80-positive, and Hsp70-positive cells were counted in 10 randomly microscopic fields at 40x objective magnification in each sample. All the IHC slides were independently and separately scored by two board-certified veterinary pathologists from NTU veterinary hospital without knowledge of any of the treatments.

### Statistical analysis

All results were analyzed using Wilcoxon-Mann-Withney test. Differences were considered statistically significant at a *P*-value of less than 0.05.

## Results

### Nano formulation of curcumin plus resveratrol enhanced the absorption in serum of rat model

First step, the oral bioavailability of the nanosized compound of curcumin plus resveratrol were analyzed in rat model. Blood levels after oral administration of nano-compound were compared with the oral original state curcumin and resveratrol suspension. The mean curcumin and resveratrol concentrations in the serum after oral administration of curcumin nanoparticles (17.85 ± 10.94 ng/mL), curcumin suspension (0.70 ± 0.62 ng/mL) at 1 h and resveratrol nanoparticles (646 ± 335.41 ng/mL), resveratrol suspension (76.5 ± 12.47 ng/mL) at 15 min at single dose in SD rats were analyzed. The AUC (0-last) value of curcumin after oral administration of curcumin nanoparticales was 215 ± 46.4 ng*hr./mL, which was 4-fold greater than that after marketed curcumin suspension administration. The Tmax value of resveratrol after oral administration of resveratrol nanoparticales was 0.83 ± 1.01 h, which was 3 fold greater than that after marketed curcumin suspension administration. The peak concentration (C_max_) and time of peak concentration (T_max_) were obtained directly from the individual plasma curcumin and resveratrol concentration versus time profiles. The area under the concentration time curve from 0 to the last measurable concentration (AUC_(0-last)_) was calculated using the trapezoidal method (10.1016/S0378-5173(98)00182-3). The AUC determines the bioavailability of the drug for a given dose of the formulation. These oral pharmacokinetic parameters are listed in Table 1.

**Table 1:** Pharmacokinetic parameters derived from rat plasma^a^

Sample	AUC_(0-last)_ (ng^a^hr./mL)	C_max_ (ng/mL plasma)	T_max_ (hr)
Curcumin suspension	46.3 ± 30.7	18.9 ± 20.1	2.5 ± 1.8
Curcumin nanoparticles	215 ± 46.4	37.7 ± 21.8	2.17 ± 1.44
Resveratrol suspension	1608 ± 284	522 ± 152	2.67 ± 0.58
Resveratrol nanoparticles	1632 ± 286	782 ± 105	0.83 ± 1.01

^a^*AUC* area under the blood concentration vs time curve;

*C*_*max*_ maximum concentration;

*T*_*max*_ time to reach C_max_

### Nano formulation of curcumin plus resveratrol inhibited the cell viability in CT26

**Fig. 2:**
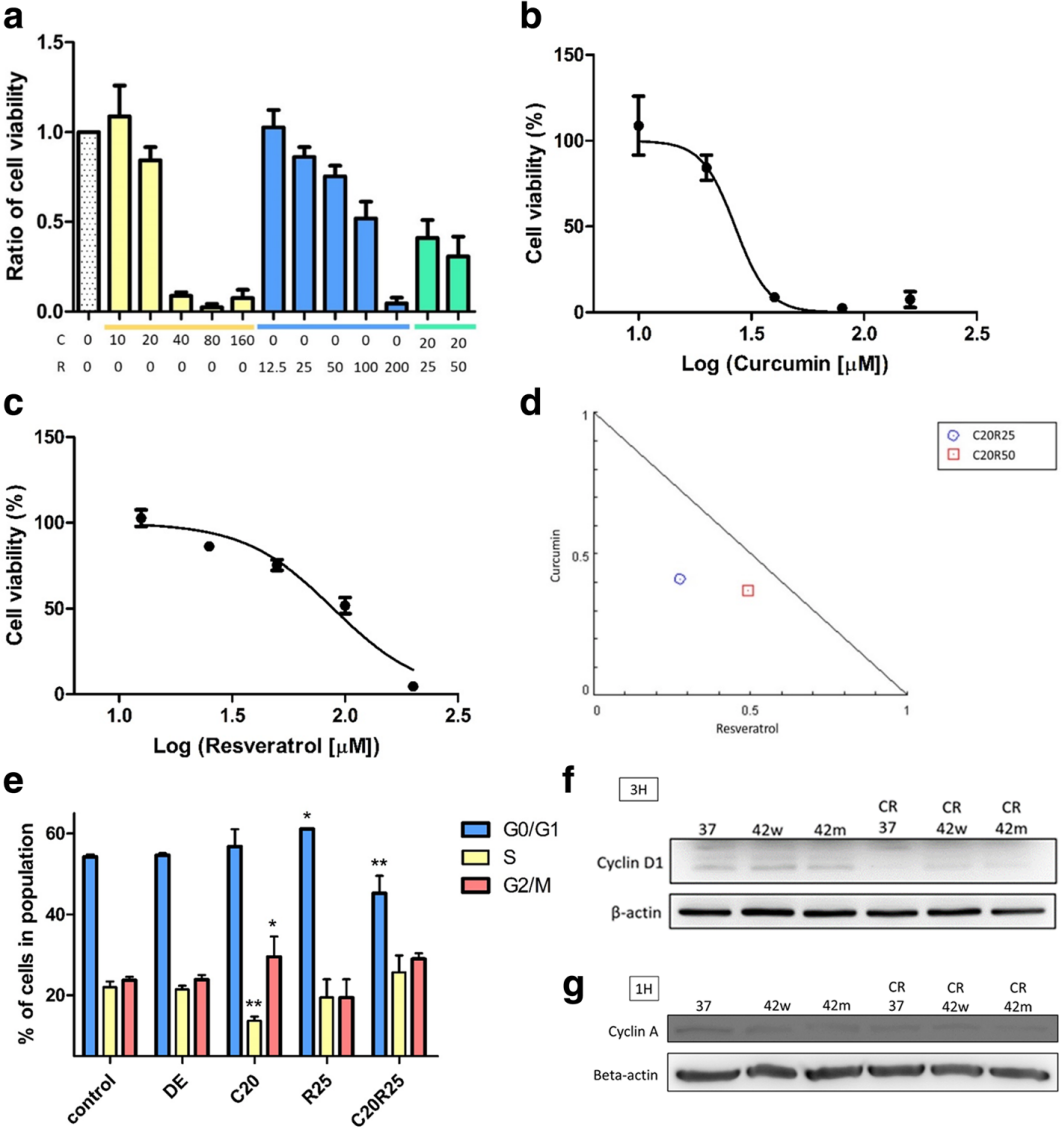
Effects of curcumin and resveratrol on the cell viability and cell cycle analysis of CT26 cells. **(a)** Cells were treated with curcumin (**c**), resveratrol (R), and the combined (C20 + R25, C20 + R50 μM) at the indicated concentration for 24 h. Cell viability was detected through WST1 assay. The results were represented the mean ± S.D. of three independent experiments. DMSO and EtOH served as the solvent control. **(b)** The cell viability of CT26 cells treated with curcumin. The IC50 of curcumin was 26.76 ± 1.06 μM. **(c)** The cell viability of CT26 cells treated with resveratrol. The IC50 of resveratrol was 88.76 ± 1.07 μM. **d.** Curcumin and resveratrol combination showed synergistically anti-tumor efficacy. **(e)** CT26 cells were treated with curcumin (20 μM) and resveratrol (25 μM) for 24 h and the cell cycle was analyzed by PI staining and flow cytometry. **(f)** Cyclin D1 and **(g)** Cyclin A expressions of CT26 cells treated by the indicated curcumin and resveratrol treatments with or w/o the 42 °C water bath (42w) and 42 °C mEHT (42 m) were analyzed by the western blot. Treating groups: DMSO + EtOH (DE, vehicle control), curcumin 20 μM (C20), resveratrol 25 μM (R25), curcumin 20 μM combined resveratrol 25 μM (C20R25). *P < 0.05, **P < 0.01 as compared to the control group. The full-length blots were presented in Supplementary Figure 2

We used WST-1 cell viability assay to detect the anti-tumor efficacy of curcumin (C) and resveratrol (R), in either single or combined use on CT26 cells. The results indicate that both curcumin and resveratrol had anti-tumor efficacy to CT26 cells in a dose-dependent manner (C: 0 ~ 160 μM; R: 12.5 ~ 200 μM). However, combined usage (C:20 + R:50 μM) dramatically decreased the cell viability at lower concentrations compared to that of single use (Fig. 2a). The IC50 of curcumin, resveratrol, and combined treatment on CT26 were 26.76 ± 1.06 and 88.76 ± 1.07 μM (Fig. 2b & c). The data suggests that curcumin and resveratrol may induce synergistically tumor inhibitory effect for CT26 cells (Fig. 2d).

### Nano formulation of curcumin plus resveratrol induced cell cycle arrest in CT26

We next investigated the mechanism of decreasing cell viability by curcumin and resveratrol. CT26 cells were treated with curcumin and resveratrol for 24 h and their cell cycle profiles were analyzed. The CT26 cells treated with curcumin (20 μM) significantly decreased S-phase ratio (13.69 ± 0.83%) while increased G2/M phase ratio (29.53 ± 4.12%) compare to control group (S-phase: 22.03 ± 1.14%, *P* = 0.005; G2/M phase: 23.74 ± 0.68%, *P* = 0.049). The CT26 cells treated with resveratrol, the G0/G1 phase ratio (61.11± 0.01%) was significantly higher than that of control group (54.23 ± 0.46%) (*P* = 0.011). These results were in concordance with the previous studies of curcumin and resveratrol on cell cycle arrest [39, 40]. Interestingly, combined treatment of curcumin and resveratrol also induced a significantly lower G0/G1 phase ratio (45.3 ± 3.45%) (*P* = 0.002) (Fig. 2e). The cell cycle alteration resulting from the treatment of curcumin and resveratrol was further confirmed by investigating the cyclins associated with G0/G1and G2/M phases. Both Cyclin D1 (Fig. 2f) and Cyclin A (Fig. 2g) decreased after CR treatment on CT26 to reveal decreased cell viability was partially due to their sabotaging cell cycle progression.

### Nano formulation of curcumin plus resveratrol with mEHT increased significant apoptosis and immunogenic cell death in CT26

**Fig. 3:**
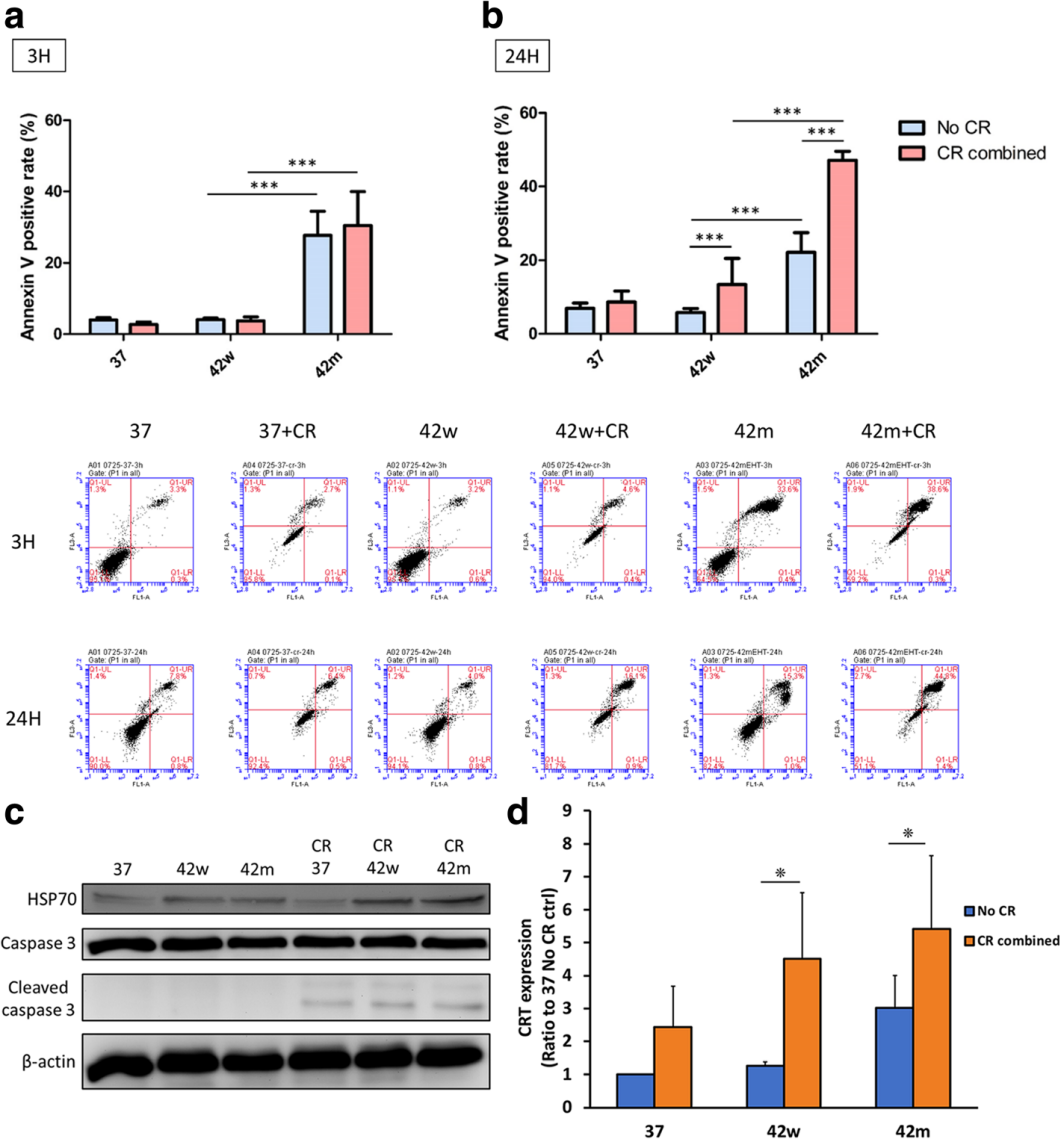
Apoptosis and immunogenic cell death of the CT26 cells treated with curcumin, resveratrol and mEHT. **(a)** CT26 cells were treated with 37 °C (37), water bath (42w), mEHT (42 m), 37 °C (37) + curcumin (C, 20 μM) and resveratrol (R, 25 μM), water bath + CR (42w + CR), and mEHT + CR (42 m + CR) for 3 or (**b**) 24 h. The apoptotic effects of these treatments were measured by annexin V/propidium iodide staining and flow cytometry. The results were represented the mean ± S.D. of three independent experiments. *P < 0.05, ***P < 0.001. **(c)** The expressions of HSP70 and caspase 3 were analyzed by western blot after CT26 cells incubated with the indicated treatments for 3 h. The full-length blots were presented in Supplementary Figure 2. **(d)** The expressions of CRT were detected by flow cytometry after CT26 cells incubated with the indicated treatments for 24 h. The results were represented the mean ± S.D. of three independent experiments. *P < 0.05

mEHT was widely used to promote the synergistic effects in a variety of cancer therapies [11, 12]. To further evaluate the anti-tumor efficacy of curcumin and resveratrol combined with mEHT, we next investigated their cell apoptotic effects using annexin V/propidium iodide staining. The 3 h treatment showed mEHT treatment (42 °C mEHT alone, 42 °C mEHT combined with curcumin and resveratrol (42 m + CR)) could induce a significantly higher apoptosis rate (Fig. 3a). After 24 h treatment, though both mEHT-treated groups showed significantly higher apoptosis rates, the 42 m + CR induced more apoptotic cells compare to that of mEHT alone (Fig. 3b). The potentially apoptosis triggering effects resulted from curcumin, resveratrol, and 42 m + CR were further confirmed by western blotting to reveal the increased cleavage form of apoptotic proteins Caspase-3 (Fig. 3c). Taken together, data indicates mEHT combined with curcumin and resveratrol can further promote the apoptosis of CT26 cells. Some dying apoptotic cells release their cellular contents and these contents contain damage-associated molecular patterns (DAMPs), including calreticulin (CRT), heat shock proteins (Hsp), high mobility group B1 (HMGB1) and other molecules, which act as danger signals to immunogenic cell death (ICD) and induce protective antitumor immunity [41]. To investigate ICD induction of curcumin and resveratrol combined with mEHT, we detected expression of Hsp70 and CRT in different treatment groups. Hsp70 protein expression was shown as hyperthermia positive control [42] and was increased in 42 m + CR group (Fig. 3c). Expression folds change of calreticulin were related to 37 °C group. Expression folds change of calreticulin increased significantly in 42 °C mEHT alone (3.02 ± 0.98) in without CR treatment groups (Fig. 3d) as shown as previously study [7]. After combination of CR treatment, expression change folds of CRT were increased in 37 °C, 42 °C and 42 °C mEHT groups (2.43 ± 1.25, 4.51 ± 2.00, 5.42 ± 2.22, respectively). These results showed curcumin and resveratrol induce cell apoptosis and immunogenic cell death to trigger further immune response.

### CT26 tumors were inhibited by nano formulation of curcumin plus resveratrol combined with mEHT treatment

**Fig. 4:**
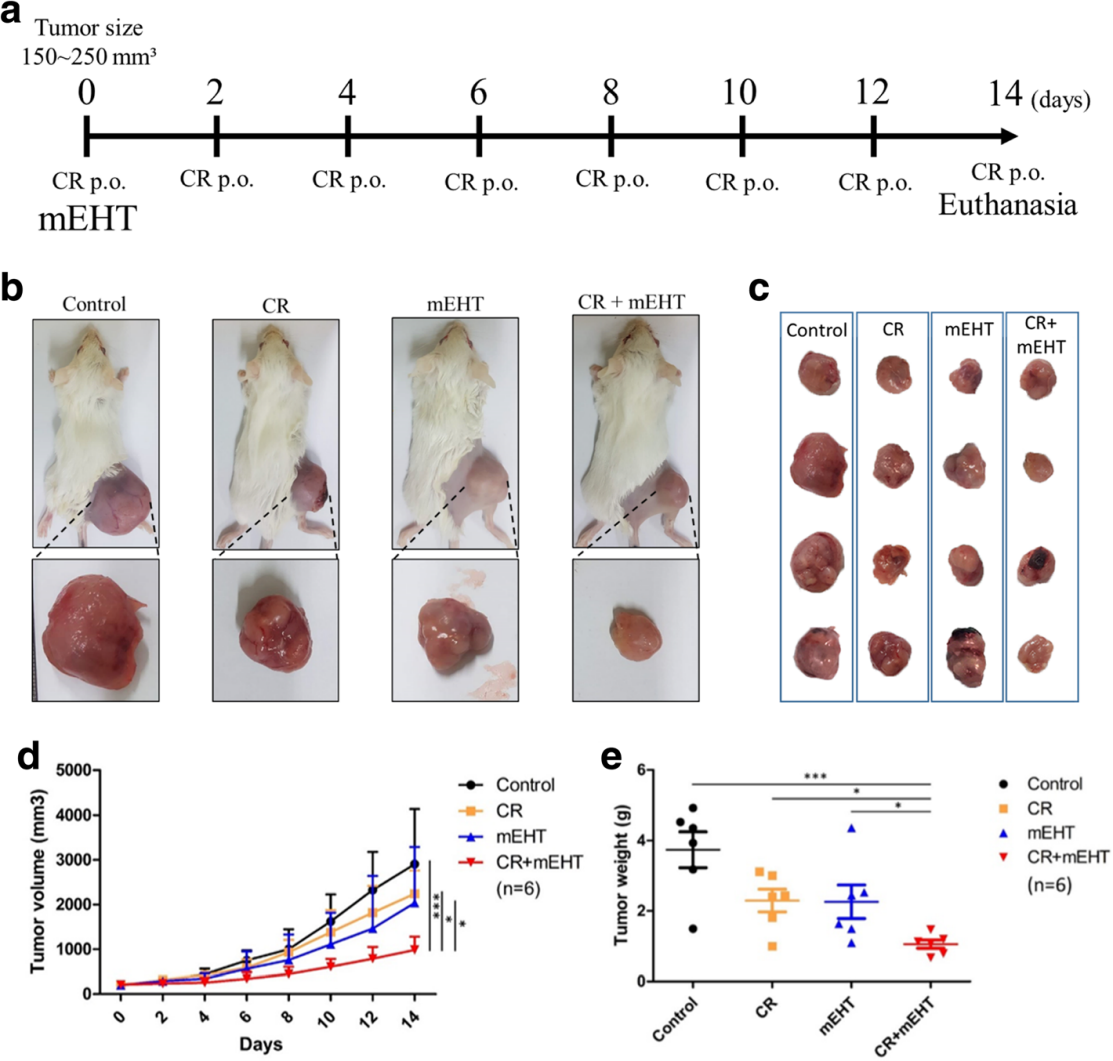
In vivo anti-tumor effect of the combined mEHT treatment with curcumin and resveratrol. **(a)** The schematic illustration of the combined treatment protocol. The tumor-bearing mice (**b**) and the tumor samples (**c**) obtained from the different treatments. The tumor growth curve (**d**) and tumor weight (**e**) of the CT26 tumors received the treatments of vehicle control (Control), curcumin (**c**), resveratrol (R), mEHT, and combined all (CR + mEHT). *P < 0.05, **P < 0.01, ***P < 0.001

The CT26 tumors established in BALB/c mice were used to evaluate the anti-cancer efficacy of curcumin and resveratrol combined with mEHT treatment. After 14 days of treatment, the mice were euthanized, and the tumors were resected to evaluat the effects induced by different treatments (Fig. 4a). The results showed that both the mean tumor volume (Fig. 4b-d) and tumor weight (Fig. 4e) of CR + mEHT group were significantly smaller and lighter than that of other groups. These results were in concordance with our in vitro findings and indicated that curcumin and resveratrol oral administration combined mEHT treatment could significantly suppress tumor growth. Additionally, temperature measured by sensors indicated that the tumor was specifically heated by the mEHT (T1) while neither the adjacent region (T2) of the tumor nor the core body temperature (T3) was elevated (Fig. 1c).

### Increased infiltration of macrophages and T-lymphocytes and Hsp70 expression were observed in tumors treated by CR and mEHT combination

**Fig. 5:**
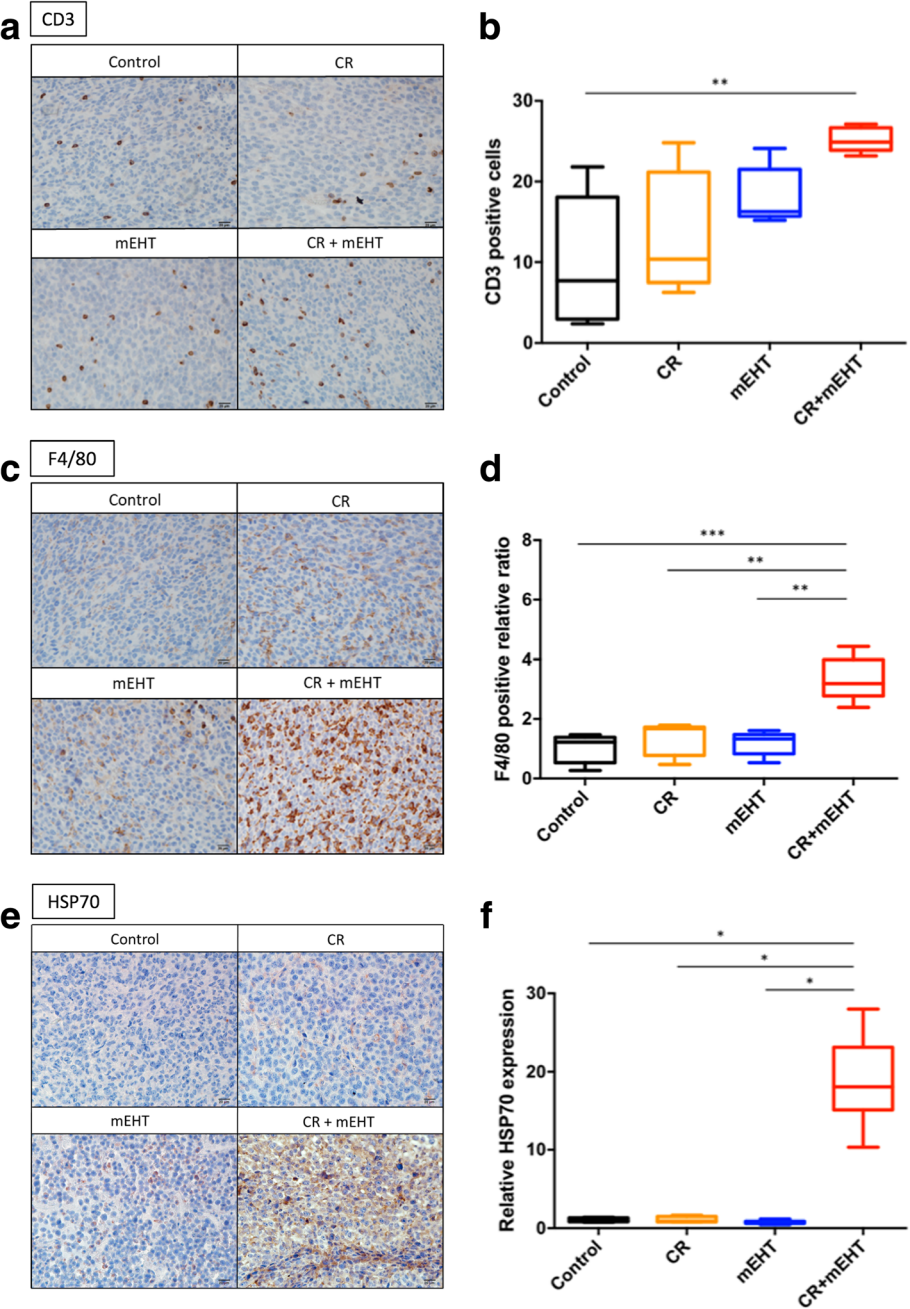
Immunohistochemical analysis of CD3, F4/80 and HSP70 expressions in tumor tissues with CR + mEHT treatment. CD3 (**a** & **b**), and F4/80 (**c** & **d**)-positive cells and HSP70 expression (**e** & **f**) within the tumor tissues treated with the indicated treatments were detected by immunohistochemistry (IHC). Vehicle control (Control), curcumin (C), resveratrol (R), mEHT, and combined all (CR + mEHT). **P < 0.01, ***P < 0.001 as compared to control group

To evaluat the immune responses potentially induced by curcumin, resveratrol, or mEHT, we used immunohistochemistry to analyze the immune cell infiltration within tumor tissues. Our data revealed that both the amounts of CD3+ T-lymphocytes (Fig. 5A & b) and F4/80+ macrophages (Fig. 5c & d) in CR + mEHT group were significantly higher than that of the Control. This indicates that in addition to reduced tumor cell viability, combined treatment of CR and mEHT could also trigger host immunity by recruiting T-cells and macrophages. Moreover, overexpression of Hsp70 was also found in tissues of CR + mEHT group (Fig. 5e & f ). Since Hsp70 is known as a danger signal induced by cell stress including hyperthermia and curcumin treatments [43] and able to attract and activate antigen-presenting cells (APCs), the results support our hypothesis that potential immune activation was induced by CR treatment and mEHT for CT26 tumor eradication.

## Discussion

Many cancer therapies are well-developed to show their efficient anti-tumor efficacy. However, most of these cancer treatments may also cause severe side effects. In this study, we demonstrated the combination of mild hyperthermia treatment with two natural antioxidants, curcumin and resveratrol, could synergistically activate host immunity and inhibit cancer development with limited side effects.

Curcumin and resveratrol are natural antioxidants with low toxicity [16, 17]. These two natural compounds have potential to increase anti-tumor efficacy by inducing tumor cell apoptosis and cell cycle arrest [22–24]. However, poor in vivo bioavailability has restricted their application in clinical usage [26, 36]. Many approaches have been applied to increase the water solubility and/or bioavailability of food bioactives by methods such as emulsion and micelle encapsulation. Additionally, chemical modification methods were also reported to increase the water solubility of curcumin, ex, liposome, and phytosomes [44]. In this study, we increased absorption rate of curcumin by physical grinding without chemical modification. There were several absorption enhancers that have also been used to improve curcumin’s bioavailability. Piperine has been found to enhance the bioavailability of curcumin both in preclinical studies and in studies on human volunteers [45]. The previous study showed that piperine can efficiently block the action of intestinal and hepatic glucuronidation enzymes, thereby increasing the bioavailability of curcuminoids [46].

Some studies had demonstrated that curcumin combined with resveratrol could achieve positive synergistic effects and inhibit tumor growth by upregulating their concentrations in serum and tissues [37]. However, the precise mechanism of the interaction between curcumin and resveratrol remains unclear. In order to improve the poor uptakes of curcumin and resveratrol within animals, we prepared their nanosized forms. The colon cancer cells CT26 were treated with nanosized compound of curcumin plus resveratrol, which revealed synergistic anti-tumor effects by inducing cell cycle arrest, apoptosis, and necrosis in vitro and in vivo. The results suggest a safe and efficient strategy for cancer therapy.

mEHT is a kind of hyperthermia which triggers apoptosis and necrosis of tumor cells by heating to 42 °C by radiofrequency [2, 6]. By specifically enabling tumor cells to absorb higher energy provided by mEHT, the temperatures of cell membrane rafts are increased, and membrane integrity is violated, enhancing cellular uptake of anti-tumor candidates [5]. mEHT has been also reported to enhance local tumor blood flow and increase the accumulation of chemotherapeutic drugs within tumor tissues [13]. In this study, we combined curcumin (C) and resveratrol (R) with mEHT treatment in vitro and in vivo. The results showed that CR + mEHT treatment significantly induced higher cell apoptosis compared to that of other groups (Fig. 3), revealing that mEHT could enhance the anti-tumor efficacy of curcumin and resveratrol.

When combining mEHT with curcumin and resveratrol, it was found to significantly inhibited CT-26 tumor development growth in BALB/c mice. The tumor volume and weights were significantly lower in CR + mEHT treatment group (Fig. 4). Moreover, the obvious increase of infiltrated F4/80+ macrophages and CD3+ T-cells were observed in the tumors receiving this treatment. Meanwhile, overexpression of Hsp70 were also found in CR + mEHT group. HSPs are highly conserved constituents of all kinds of prokaryotic and eukaryotic cells, which are known as intracellular chaperone proteins associated with cell stress [47]. The intracellular and inducible HSPs may turn immunogenic when complexed with tumor peptides [48] and HSPs were also found outside the cells or located at the tumor cell surface. In our in vitro study, the CR + mEHT group showed the highest intracellular Hsp70 protein expression and highest apoptosis rate. This increased apoptosis and necrosis leads to form tumor peptides, and can be complexed by HSPs to become HSP-chaperoned peptides. Thus, APCs could utilize the uptake of HSP-chaperoned peptides for the loading of MHC Class I molecules and thus stimulate a specific T-cell response [49, 50]. Our data might have supported this HSP-mediated APC recruiting mechanism since significantly higher T-lymphocyte and macrophage infiltration were found in CR + mEHT group.

## Conclusions

In summary, this study indicates that nano-formulated curcumin plus resveratrol compound shows enhanced bioavailability when combined with mEHT, synergistically increasing HSP-release and immune response, leading to enhanced anti-tumor efficacy in CT26 tumors. This study suggests this treatment is safe. However, further clinical studies are needed to confirm the safety and effectiveness of nano-formulated curcumin and resveratrol when combined with mEHT.

## Supplementary information

**Additional file 1 Supplementary Figure 1*.*** In vitro mEHT instrument. CT26 cells (2 × 10^6^) were contained within the cell bag which was settled in the electrode chamber. (A) The cells were then heated at 42 °C for 30 min. The optical sensors were used to detect the temperature within the cell bag (T1) or electrode chamber (T2). Left and middle were the schematic diagrams while right showed the in vitro mEHT device. (B) The whole mEHT in vitro device. **Supplementary Figure 2** (Original blots for the figures).

## Data Availability

The datasets used and/or analyzed during the current study are available from the corresponding author on reasonable request.

## Abbreviations

C: Curcumin
R: Resveratrol
CR: Curcumin combined Resveratrol
mEHT: Modulated electro-hyperthermia
HSPs: Heat shock proteins
APCs: Antigen presenting cells
