# Supplementary material for: Potential enhancement of host immunity and anti-tumor efficacy of nanoscale curcumin and resveratrol in colorectal cancers by modulated electro- hyperthermia
Source: BMC Cancer. 2020 Jun 29;20:603. doi: 10.1186/s12885-020-07072-0 (PMC7324975; doi:10.1186/s12885-020-07072-0)
Supplement: Supplementary file 1 — Additional file 1 Supplementary Figure 1. In vitro mEHT instrument. CT26 cells (2 × 10^6^) were contained within the cell bag which was settled in the electrode chamber. (A) The cells were then heated at 42 °C for 30 min. The optical sensors were used to detect the temperature within the cell bag (T1) or electrode chamber (T2). Left and middle were the schematic diagrams while right showed the in vitro mEHT device. (B) The whole mEHT in vitro device. Supplementary Figure 2 (Original blots for the figures). [file 12885_2020_7072_MOESM1_ESM.docx]

**Supplementary Figure 1**

**A
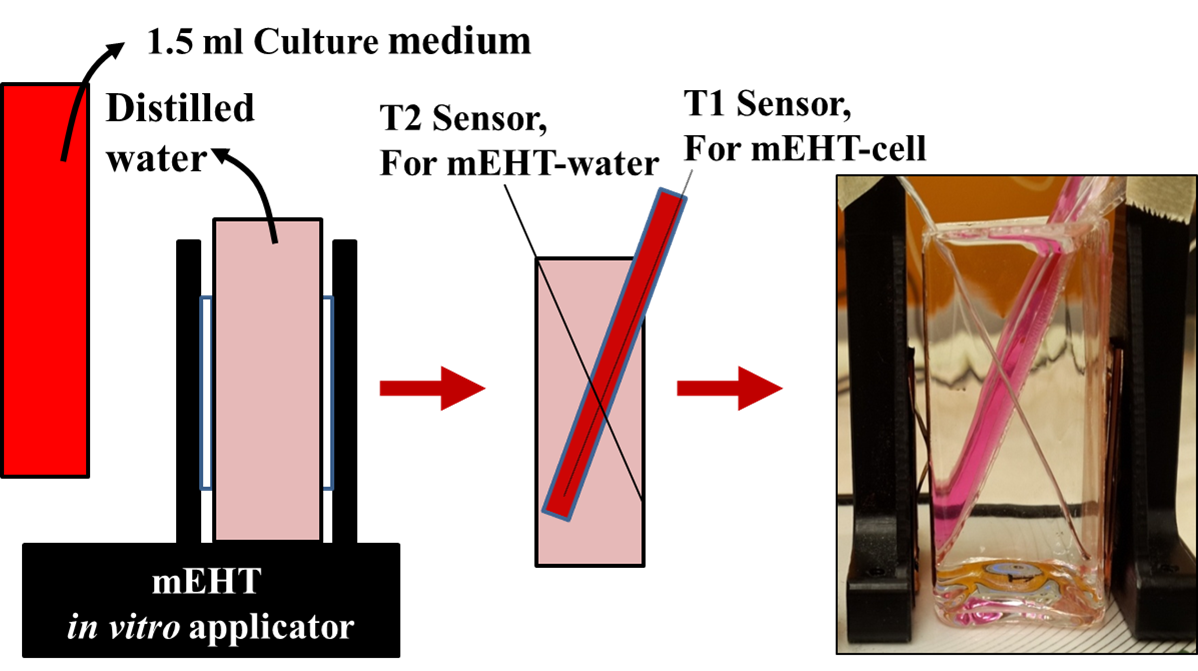
**

*in vitro*

**Electrode chamber**

**Cell bag**

**CT26**

**B**


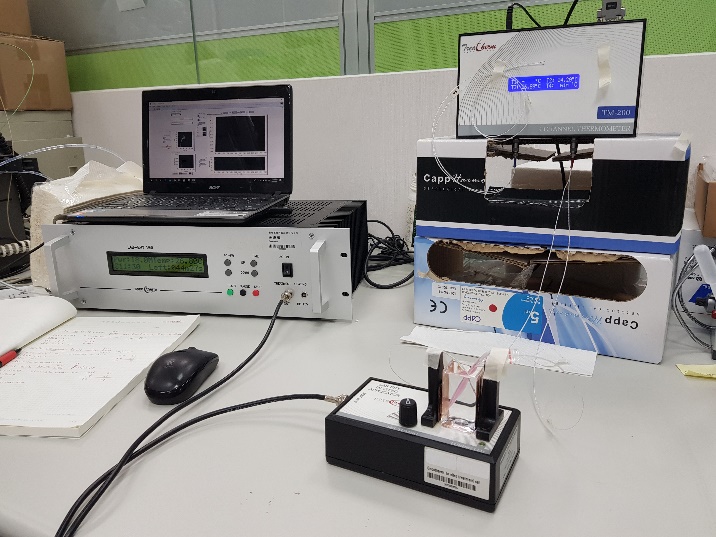


***Supplementary Fig. 1.*** *In vitro* mEHT instrument. CT26 cells (2 × 10^6^) were contained within the cell bag which was settled in the electrode chamber. (A) The cells were then heated at 42 °C for 30 min. The optical sensors were used to detect the temperature within the cell bag (T1) or electrode chamber (T2). Left and middle were the schematic diagrams while right showed the *in vitro* mEHT device. (B) The whole mEHT in vitro device.

**Supplementary Figure 2 (Original blots for the figures)**

Fig. 2F Cyclin D1

**
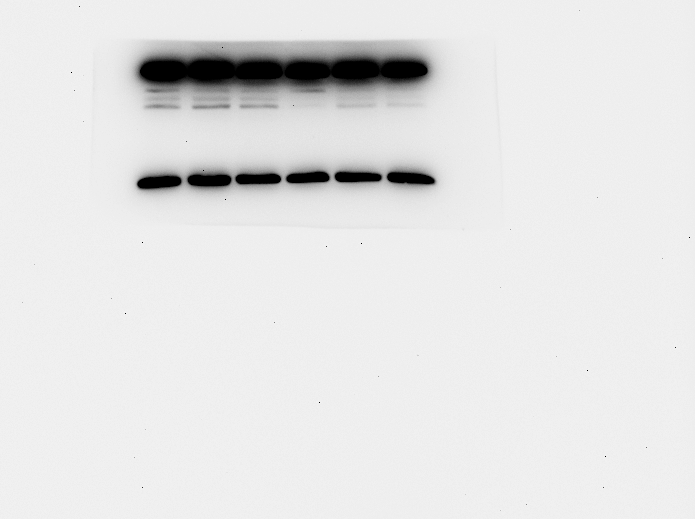
**

Fig. 2F β-actin

**
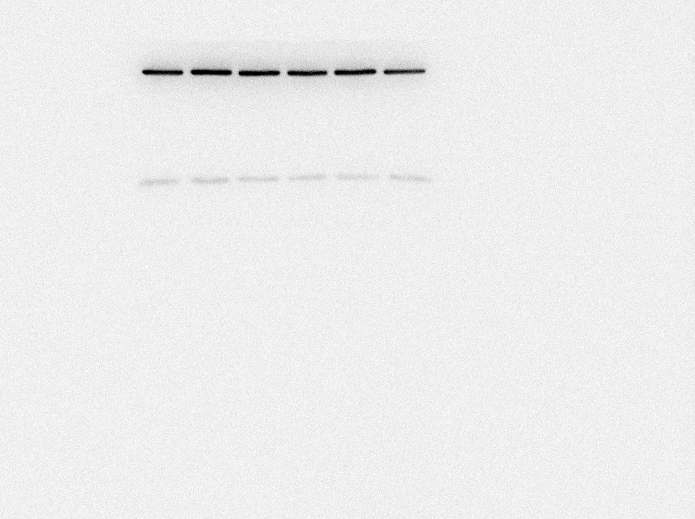
**

Fig. 2G Cyclin A

**
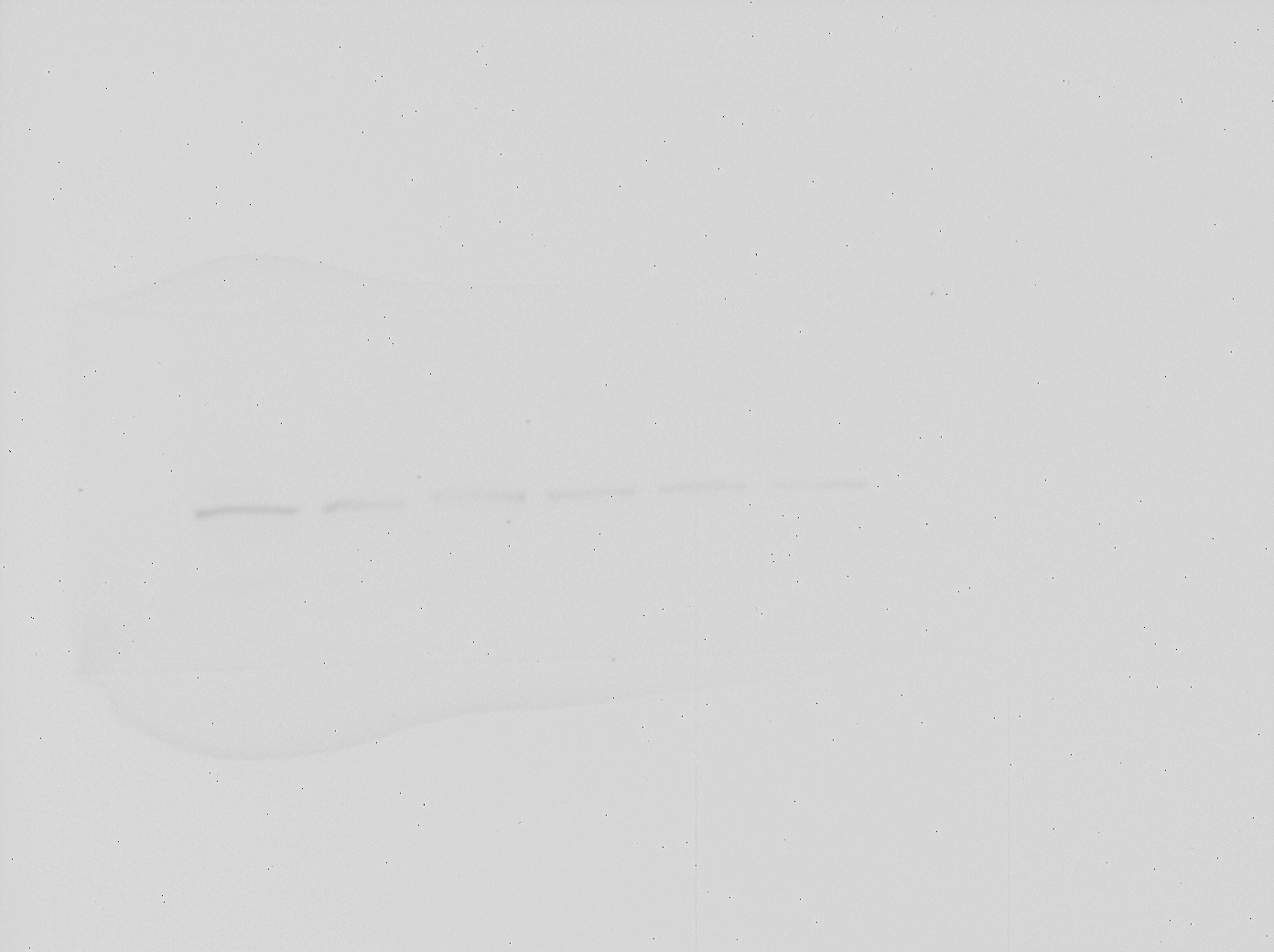
**

Fig. 2G β-actin

**

**

Fig. 3C HSP70

**
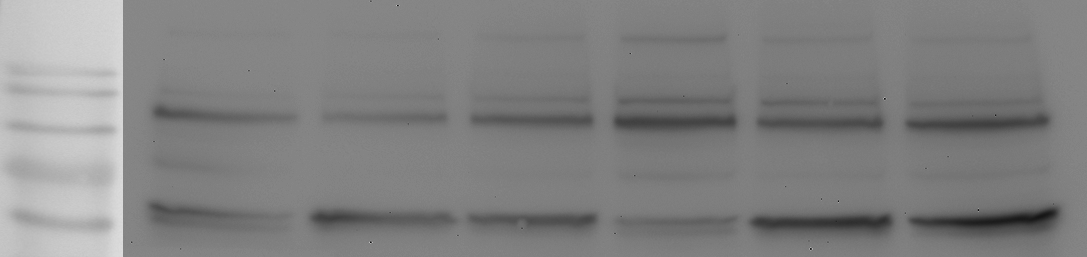
**

Fig. 3C β-actin , Caspase 3 & Cleaved Caspase 3

**
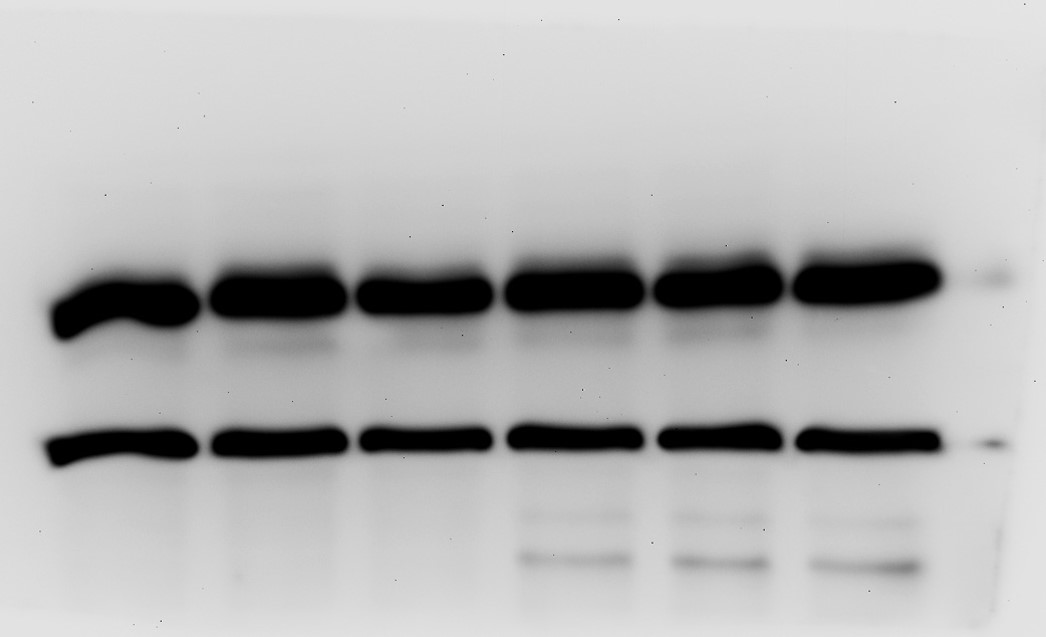
**

Cleaved

caspase 3

Caspase 3

β-actin
